# Betulinic Acid Suppresses Breast Cancer Metastasis by Targeting GRP78-Mediated Glycolysis and ER Stress Apoptotic Pathway

**DOI:** 10.1155/2019/8781690

**Published:** 2019-08-19

**Authors:** Yifeng Zheng, Pengxi Liu, Neng Wang, Shengqi Wang, Bowen Yang, Min Li, Jianping Chen, Honglin Situ, Meiqi Xie, Yi Lin, Zhiyu Wang

**Affiliations:** ^1^Integrative Research Laboratory of Breast Cancer, The Research Centre of Integrative Medicine, Discipline of Integrated Chinese and Western Medicine & The Second Affiliated Hospital of Guangzhou University of Chinese Medicine, Guangzhou, 510006 Guangdong, China; ^2^Guangdong Provincial Key Laboratory of Clinical Research on Traditional Chinese Medicine Syndrome, Guangdong Provincial Academy of Chinese Medical Sciences, Guangdong Provincial Hospital of Chinese Medicine, Guangzhou, 510006 Guangdong, China; ^3^Post-Doctoral Research Center, Guangzhou University of Chinese Medicine, Guangzhou, 510006 Guangdong, China; ^4^College of Basic Medicine, Guangzhou University of Chinese Medicine, Guangzhou, 510006 Guangdong, China; ^5^School of Chinese Medicine, Hong Kong Baptist University, SAR, Hong Kong 999077, China; ^6^School of Chinese Medicine, The University of Hong Kong, SAR, Hong Kong 999077, China

## Abstract

Targeting aberrant metabolism is a promising strategy for inhibiting cancer growth and metastasis. Research is now geared towards investigating the inhibition of glycolysis for anticancer drug development. Betulinic acid (BA) has demonstrated potent anticancer activities in multiple malignancies. However, its regulatory effects on glycolysis and the underlying molecular mechanisms are still unclear. BA inhibited invasion and migration of highly aggressive breast cancer cells. Moreover, BA could suppress aerobic glycolysis of breast cancer cells presenting as a reduction of lactate production, quiescent energy phenotype transition, and downregulation of aerobic glycolysis-related proteins. In this study, glucose-regulated protein 78 (GRP78) was also identified as the molecular target of BA in inhibiting aerobic glycolysis. BA treatment led to GRP78 overexpression, and GRP78 knockdown abrogated the inhibitory effect of BA on glycolysis. Further studies demonstrated that overexpressed GRP78 activated the endoplasmic reticulum (ER) stress sensor PERK. Subsequent phosphorylation of eIF2*α* led to the inhibition of *β*-catenin expression, which resulted in the inhibition of c-Myc-mediated glycolysis. Coimmunoprecipitation assay revealed that BA interrupted the binding between GRP78 and PERK, thereby initiating the glycolysis inhibition cascade. Finally, the lung colonization model validated that BA inhibited breast cancer metastasis *in vivo*, as well as suppressed the expression of aerobic glycolysis-related proteins. In conclusion, our study not only provided a promising drug for aerobic glycolysis inhibition but also revealed that GRP78 is a novel molecular link between glycolytic metabolism and ER stress during tumor metastasis.

## 1. Introduction

Breast cancer is the most diagnosed malignancy among women worldwide. In 2018, it is estimated that 2.1 million new cases will be diagnosed. Breast cancer accounts for cancer occurrences in almost 1 in 4 females and for 11.6% of all sites of malignancies in both men and women [1]. Importantly, breast cancer is also the leading cause of cancer deaths among women in over 100 countries. There will be 62679 breast cancer deaths globally in 2018, which accounts for 6.6% of all site cancer deaths in both sexes [2, 3]. According to cancer death cause analysis, metastasis is always the leading reason and tremendous endeavor has been dedicated to its underlying mechanisms, such as cancer stem cells, immune depression, and metabolic alteration. However, candidate drugs approved for the inhibition of metastasis are very limited, and natural phytochemicals have become an important resource to discover precursors of metastasis inhibitors.

Metabolic reprogramming is one of the hallmarks of cancer [4], especially for aerobic glycolysis. Since the first report of cancer glycolysis activity by Otto Warburg in 1920, several studies have demonstrated that cancers prefer glycolysis even in the presence of oxygen, a phenomenon known as “Warburg effect” [5, 6]. At present, the Warburg effect of tumor could be monitored by 18F-fluorodeoxyglucose positron-emission tomography (FDG-PET) to indicate metastasis information [7]. At the same time, a number of molecular targets have been identified in the glycolysis pathway and been paid with great interests for metastasis inhibition and anticancer drug development. For example, lactate dehydrogenase A (LDHA) was found to promote breast cancer metastasis and its inhibitor oxamate was effective in inhibiting cancer cell invasion in multiple malignancies [8]. Pyruvate dehydrogenase kinase 1 (PDK1) overexpression could enhance head and neck squamous carcinoma metastasis via the upregulation of fibronectin [9]. The hexokinase II inhibitor 2-DG or 3-BrPA had the inhibitory effect on tumorigenesis and metastasis in multiple malignancies such as lung cancer, liver cancer, and breast cancer [10, 11]. Consequently, glycolysis inhibition may be a promising new strategy for antimetastasis. However, the internal mechanisms of aerobic glycolysis have not been fully elucidated. Identification of prime carcinogenic signaling is critical for the development of glycolysis inhibition strategy. Recent studies suggested that glucose-regulated protein (GRP78) serves as a molecular hub in mediating metabolism regulation and cancer metastasis [12].

GRP78, a major chaperone in the endoplasmic reticulum, is a central sensor of cellular stress and is frequently highly expressed in most solid tumors [13]. High expression of GRP78 contributes to the acquisition of metastatic phenotypes including apoptosis resistance, immune escape, angiogenesis, and drug resistance [12]. It has been reported that GRP78 is involved in the development of metastatic breast cancer as a multifunctional receptor when it is expressed on the cancer cell surface [14]. In fact, GRP78 also participates in cancer cell metabolism regulation. Glucose deficiency usually leads to GRP78 overexpression, which enhances glutamine metabolism to support cell survival by modulating *β*-catenin signaling [15]. A recent report demonstrated that GRP78 regulated metabolic reprogramming by modulating acetyl-CoA production and histone acetylation in prostate cancer cells [16]. In addition, another study indicated that GRP78 induction could result in enhanced *HIF-1a* transcription and GLUT1 expression, which are the key factors contributing to glycolysis [17]. Given the membrane translocation of GRP78 under cellular stress and its biofunction in controlling glycolysis and metastasis, it is interesting and promising to develop candidate inhibitors targeting GRP78 from natural phytochemicals, which may overcome the limitations of existing glycolytic inhibitors. For example, although 2-deoxyglucose and 3-bromopyruvic acid showed excellent anticancer effects in preclinical studies, their clinical applications were significantly limited due to the serious systemic adverse effects [18]. Therefore, the demand for developing a glycolysis inhibitor with high safety is highly appreciated.

BA, a pentacyclic triterpene widely found in birch bark extracts, has been reported to act anticancer activities in multiple cancers, including breast cancer [19]. What is more important, it was found that BA did not display apparent systemic toxicity in tumor-bearing mice even at 500 mg/kg [20]. Subsequent studies also suggested that BA did not exhibit discernable impact on normal cells at doses which killed cancer cells *in vitro* [21]. Therefore, BA attracts increasing attention due to its high selectivity for cancer cells. With regard to pharmacological mechanisms, current findings include (i) the induction of cancer cell apoptosis via the mitochondrial pathway induced by the release of soluble factors or generation of reactive oxygen species (ROS) [22, 23]; (ii) the inhibition of angiogenesis [24]; (iii) the degradation of transcription factor specificity protein 1 (Sp1) [25, 26]; and (iv) the induction of DNA damage by suppressing topoisomerase I [27, 28]. Notably, a recent report suggested that BA could change cellular glucose metabolism with concomitant reduction of glucose oxidation [29]. Besides, we also noticed that BA exerted antimetastatic potential by reversing EMT in melanoma cells via repressing the expression of neutrophil gelatinase-associated lipocalin (NGAL) [30]. However, the underlying molecular mechanisms of BA are far away from full elucidation. It is interesting to identify the molecular target of BA and the association with glycolysis regulation.

In the present study, we found that BA could attenuate migration and invasion of highly aggressive breast cancer cells via aerobic glycolysis inhibition. GRP78 silencing blocked the inhibitory effects of BA on glycolytic proteins including LDHA, PDK1, and c-Myc. Exploration of the molecular mechanism indicated that BA interrupted the binding between GRP78 and PERK, which subsequently activated eIF2*α* phosphorylation, and suppressed downstream signaling by *β*-catenin/c-Myc. *In vivo* studies also demonstrated that BA inhibited lung colonization of breast tumor. Our results provide novel insights of BA as a promising molecular inhibitor of breast cancer metastasis *via* glycolysis inhibition and also reveal a novel regulatory pathway between GRP78 and glycolytic metabolism in cancer cells.

## 2. Materials and Methods

### 2.1. Cell Culture

Breast cancer cell lines MDA-MB-231 and BT-549 and mammary epithelial cell line HBL-100 were purchased from the American Type Culture Collection (ATCC). The cells were cultured in the basal medium supplemented with 10% fetal bovine serum and 1% penicillin and streptomycin in a humidified incubator with 5% CO_2_ at 37°C.

### 2.2. Cell Viability

Cell viability was detected by CCK-8 assay. MDA-MB-231, BT-549, and HBL-100 were plated at a density of 3 × 10^3^ cells into the 96-well plate. After attachment, the cells were treated by serial concentration gradients of BA (Xi'an Natural Field Bio-Technique Co. Ltd., Xi'an, China) for 24 h, 48 h, and 72 h. The cell viability was measured by the CCK-8 reagent (Beyotime Biotechnology, Shanghai, China) according to the absorbance value. Three independent repetitive experiments were conducted.

### 2.3. Colony Formation Assay

1000 cells were plated into each well of a 6-well plate to disperse homogeneously.

Cells were firstly treated by 20 or 40 *μ*M BA for 4 h and then cultured with fresh medium for 2 weeks. The ultimately formed colonies were fixed with 4% paraformaldehyde and stained with Coomassie blue.

### 2.4. Wound Healing and Transwell Invasion Assay

For the wound healing assay, cells were seeded into the 6-well plate at a density of 4 × 10^5^. When the cells grew to 100% confluence, a “wound” in a cell monolayer was created and its distance was compared at 0, 12, 24, and 48 h to quantify the migration rate of the cells with or without BA treatment. To exclude the antiproliferative effects of BA on cell migration, the MDA-MB-231 and BT-549 cells were treated with BA for 12 h before scratching. After removing BA, the same amounts of cells were then cultured in serum-free medium to avoid the influence of proliferation. For Transwell invasion assays, the chambers were coated with a layer of Matrigel prior to the experiment. Similarly, MDA-MB-231 and BT-549 cells were pretreated by BA for 12 h, then quantified and seeded into the upper compartment with 200 *μ*l serum-free media (50000 cells per well). In contrast, the lower compartment contained 10% FBS. After 24 h incubation, the cells that penetrated the filter were fixed with 4% paraformaldehyde, followed by 0.1% Coomassie blue staining for 20 min.

### 2.5. TUNEL Analysis

MDA-MB-231 and BT-549 were treated with BA 20 and 40 *μ*M for 48 h. Then, the cell apoptosis was detected in situ by fluorescence using TUNEL analysis as described previously [31].

### 2.6. Western Blotting Analysis

Equal amounts of protein lysates (50 *μ*g) were loaded for SDS-PAGE and transferred to a PVDF membrane (Millipore, Billerica, MA). The signals were probed with primary antibodies and amplified by the secondary antibodies. The primary antibodies included E-cadherin antibody (20874-1-AP, Proteintech, Rosemont, IL, USA), N-cadherin antibody (22018-1-AP, Proteintech, Rosemont, IL, USA), vimentin antibody (10366-1-AP, Proteintech, Rosemont, IL, USA), MMP-2 antibody (A6247, ABclonal Technology Cambridge, Boston, USA), MMP-9 antibody (sc-13520, Santa Cruz Biotechnology, Santa Cruz, CA, USA), *β*-actin antibody (4970, Cell Signaling Technology, Danvers, MA, USA), *β*-catenin antibody (51067-2-AP, Proteintech, Rosemont, IL, USA), c-Myc antibody (A1309, ABclonal Technology Cambridge, Boston, USA), LDHA antibody (3582, Cell Signaling Technology, Danvers, MA, USA), LDHB antibody (sc-100775, Santa Cruz Biotechnology, Santa Cruz, CA, USA), PDK-1 antibody (sc-293160, Santa Cruz Biotechnology, Santa Cruz, CA, USA), p-PDK-1 antibody (3061, Cell Signaling Technology, Danvers, MA, USA), GRP78 antibody (11587-1-AP, Proteintech, Rosemont, IL, USA), caspase-12 antibody (55238-1-AP, Proteintech, Rosemont, IL, USA), CHOP antibody (15204-1-AP, Proteintech, Rosemont, IL, USA), PERK antibody (5683, Cell Signaling Technology, Danvers, MA, USA), p-PERK antibody (DF7576, Affinity Biosciences, Cincinnati, OH, USA), eIF2*α* (11233-1-AP, Proteintech, Rosemont, IL, USA), and p-eIF2*α* (AP0635, ABclonal Technology Cambridge, Boston, USA). Finally, the bands were imaged though the ECL Advance reagent (Tanon Science & Technology, Shanghai, China) and quantified by optical densities using the ImageLab software (Bio-Rad, Hercules, CA).

### 2.7. Gelatin Zymography

Cells were cultured in the 6-well plate in 10% fetal bovine serum (FBS) with or without BA treatment. At 70-80% confluence, the FBS was removed and continue to grow cells in FBS-free media. After 48 h, the conditioned media were centrifuged and collected. Adjust conditioned media in all samples to the same protein concentration at 10 *μ*g/ml before SDS-PAGE. The 7.5% acrylamide gel contained 8 mg gelatin. The gel was washed 2 × 30 min with washing buffer (2.5% Triton X-100, 50 mM Tris-HCl, pH 7.5, 5 mM CaCl_2_, and 1 *μ*M ZnCl_2_) after electrophoresis and then incubated in an incubation buffer (1% Triton X-100, 50 mM Tris-HCl, pH 7.5, 5 mM CaCl_2_, and 1 *μ*M ZnCl_2_) for 24 h at 37°C. Finally, the gel was stained with 0.5% Coomassie blue solution and in turn destained until bands can clearly be seen.

### 2.8. RT-qPCR Analysis

Total RNA was extracted with RNAiso Plus Reagent (Takara BIO, Japan) and transcribed to complementary DNA in reverse using the reverse transcription reagent kit with gDNA eraser (Takara BIO, Japan). The RT-PCR was performed by Applied Biosystems ViiA7 Real-Time PCR System (Thermo Fisher Scientific, Hudson, USA) using SYBR® Premix Ex TaqTM II kit (Takara BIO, Japan) in accordance with the manufacturer's instruction. The relative mRNA levels were compared using the 2^-*ΔΔ*Ct^ method.

### 2.9. Lactate Production Assays

Cells were treated with gradient concentration of BA for 48 h and then lysed in lactate assay buffer using VCX105 ultrasonic cell crusher (SONICS, USA). The lactate production in cell lysates was measured using the Lactate Assay Kit (Sigma-Aldrich, Shanghai, China) according to the manufacturer's instructions.

### 2.10. Cell Energy Phenotype Analysis

The cell energy phenotype profiles were analyzed though the oxygen consumption rate (OCR) and extracellular acidification rate (ECAR) values that were obtained by the Seahorse XF24 extracellular flux analyzer (Seahorse Bioscience). Briefly, 4 × 10^4^ cells per well were seeded into XF24 cell culture microplates and cultured overnight. Meanwhile, the XF24 cartridge was equilibrated with the calibration solution overnight at 37°C. On the second day, cells were treated with 40 *μ*M BA for 3 h prior to the measurement. XF assay medium (containing 10 mM glucose, 2 mM glutamine, and 1 mM pyruvate in XF base medium, pH = 7.4) was used to prepare the cellular stress-inducing reagents, including 1.0 *μ*M oligomycin, 1.0 *μ*M carbonyl cyanide 4-(trifluoromethoxy) phenylhydrazone (FCCP), 0.5 *μ*M antimycin A, and 0.5 *μ*M rotenone (final concentration). All the reagents were loaded in the ports according to the manufacturer's instructions. After the measurement, cell numbers in each well were counted and were used to normalize the OCR and ECAR values.

### 2.11. DARTS

The molecular target of BA was identified by the DARTS strategy according to the protocol provided by Lomenick et al. [32] and improved by ourselves [33]. Briefly, the breast cancer cell lines MDA-MB-231 or BT-549 were treated with gradient concentrations of BA (0.1-100 *μ*M) or DMSO control for 3 h. The cells were then lysed with protease and phosphatase inhibitors and diluted to the same concentration of protein. Each sample was proteolyzed at 4°C for 30 min with 0.05 mg/ml pronase (Roche Diagnostics, Indianapolis, USA). To find the protected bands, SDS-PAGE was applied, and the gels were stained with Coomassie blue. Protected bands were ultimately cut out and digested by trypsin for mass spectrometry analysis.

### 2.12. Plasmid Construction and Transfection

shRNAs were purchased from GenePharma (Shanghai, China), and recombinant plasmids of GRP78 were obtained from Vigene Biosciences (Maryland, USA). The scrambled plasmids and empty vector were used as control. MDA-MB-231 and BT-549 cells were transfected with lipofectamine 3000 (Invitrogen, Carlsbad, CA, USA) according to the manufacturer's protocol. The protein expressions were verified by western blotting after transfection of 48 h.

### 2.13. Coimmunoprecipitation Analysis

Coimmunoprecipitation assay was carried out by the Pierce® Co-Immunoprecipitation Kit (Thermo Fisher Scientific, Hudson, NH, USA) according to the manufacturer's instructions. In brief, GRP78 antibody was immobilized with resin. Then, the immobilized resin was incubated with the MDA-MB-231 and BT-549 cell lysates for detection of target protein PERK by immunoblotting.

### 2.14. Mouse Procedures

Five-week-old female Balb/c nude mice were obtained from the Beijing Vital River Laboratory Animal Technology Co. Ltd. Experimental treatments of all mice were reviewed and approved by the supervision of the Institutional Animal Research Ethics Committee in Guangzhou University of Chinese Medicine (Approval No. 20180912013). The mice were fed in the specific pathogen-free ventilation chambers under an ambient temperature of 20-25°C and 45-50% relative humidity and given sterilized food and water. To establish the lung colonization model of breast cancer in mice, luciferase gene-tagged MDA-MB-231 cells were injected through the tail vein at the density of 2 × 10^5^ once a week and continuing for 6 weeks. Starting from the third week, the mice were segregated into 3 groups randomly (*n* = 6), including vehicle (0.5% CMC-Na) and BA 125 and 250 mg/kg. The dosage of BA is rationalized according to previous studies [20]. BA was dissolved in DMSO and then dispersed in 0.5% CMC-Na. The final amount of DMSO was less than 5%. BA was administered by intraperitoneal injection every other day for 4 weeks. At the end of treatment, the mice were anesthetized by isoflurane inhalation and injected intraperitoneally with D-luciferin (PerkinElmer, Boston, USA) at 150 mg/kg for luminescent imaging. We imaged photonic emission with the IVIS-spectrum system (PerkinElmer, Boston, USA) and quantified bioluminescence of the lung colonization.

### 2.15. Immunohistochemistry and Hematoxylin-Eosin Staining

Tumor specimens were fixed in 4% paraformaldehyde for 24 h, followed by the protocol as we described previously [31]. Hematoxylin-eosin staining was conducted using the Hematoxylin and Eosin Staining Kit (Beyotime Biotechnology, Shanghai, China) according to the manufacturer's instructions.

### 2.16. Immunofluorescence Analysis

The lung tissue specimens were processed same as the immunohistochemistry assays and then permeabilized with 0.25% Triton X-100, following being blocked with 5% bovine serum albumin (Sigma-Aldrich, Shanghai, China) for 30 min at room temperature. Afterwards, the specimens were incubated with primary antibodies overnight at 4°C and fluorescence-conjugated secondary antibody for 1 h at room temperature in the dark. The nucleus was stained by DAPI (Sigma-Aldrich, Shanghai, China) for 20 min at room temperature. In the end, the fluorescence was visualized by the LMS710 confocal microscope (ZEISS, Jena, Germany).

### 2.17. Statistical Analysis

All statistical analyses were performed using Statistical Product and Service Solutions (SPSS) 20.0 software. The one-way ANOVA and the Dunnett post hoc test were performed for comparison among multiple groups. ANOVA for repeated measurement was performed towards repeated measures data. *P* < 0.05 was considered as statistically significant.

## 3. Results

### 3.1. BA Inhibits Metastasis of Highly Aggressive Breast Cancer Cells

**Figure 1:**
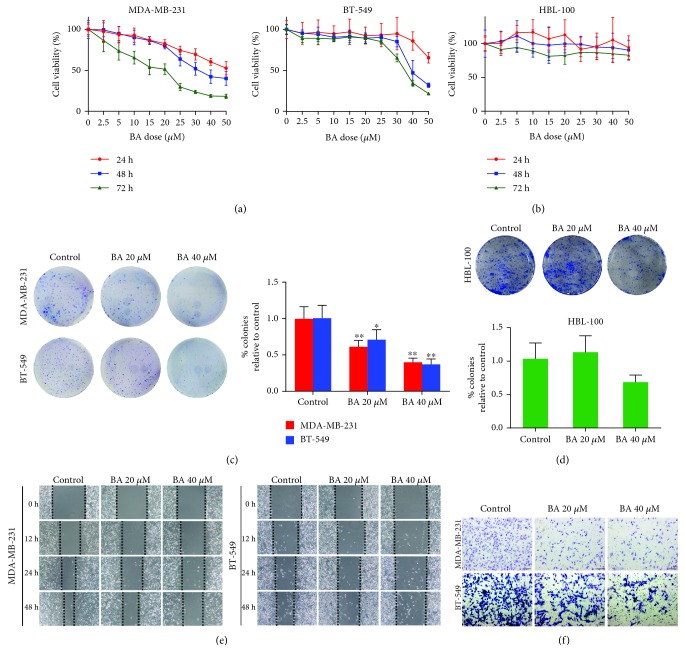
BA inhibits breast cancer cell proliferation and metastasis. (a) BA inhibited MDA-MB-231 and BT-549 cell viability in a dose- and time-dependent manner. (b) BA exerted minimal inhibitory effects on HBL-100. (c, d) 20 and 40 *μ*M BA significantly suppressed colony growth of both MDA-MB-231 and BT-549, while it did not apparently affect the colony formation of HBL-100. (e) BA significantly slowed down the confluence of wound healing, revealing its ability of migration resistance. (f) Transwell assay indicated that the number of invasive cells was reduced by BA (the results were obtained from triplicate experiments and were represented as mean values ± SD; ^∗^*P* < 0.05 and ^∗∗^*P* < 0.01 as compared with control).

Our previous study had shown that BA suppressed glycolysis metabolism of breast cancer cells [34]. Moreover, emerging evidence implied that targeting tumor cell glycolysis may be a promising strategy to inhibit metastasis. To investigate the activity of BA against breast cancer metastasis, two basal-like highly aggressive breast cancer cell lines MDA-MB-231 and BT-549 were treated by BA for 24, 48, and 72 h. The viability of both cell lines was inhibited in a time- and dose-dependent manner (Figure 1(a)). However, BA had a minimal influence on the proliferation of the nonmalignant mammary epithelial cell line HBL-100 from 24 to 72 h, confirming its highly selective inhibitory effect on malignant cells (Figure 1(b)). We next performed colony formation assays to evaluate the long-term inhibitory effects of BA. Obviously, colonies of MDA-MB-231 and BT-549 cells were significantly suppressed by BA treatment (Figure 1(c)). In contrast, BA only had a modest effect on the colony growth of HBL-100, further validating the high safety profile of BA over a long exposure period (Figure 1(d)). Based on these results, the influence of BA on cancer cell migration and invasion was analyzed by wound healing and Transwell migration assays. We found that the extent of wound healing was impaired (Figure 1(e)) and the number of invading cells passing through the membrane was significantly reduced following BA treatment (Figure 1(f)). These findings suggested that BA may also have the effect of inhibiting breast cancer metastasis, in addition to glycolysis suppression.

### 3.2. BA Blocks Breast Cancer EMT and MMP Secretion

**Figure 2:**
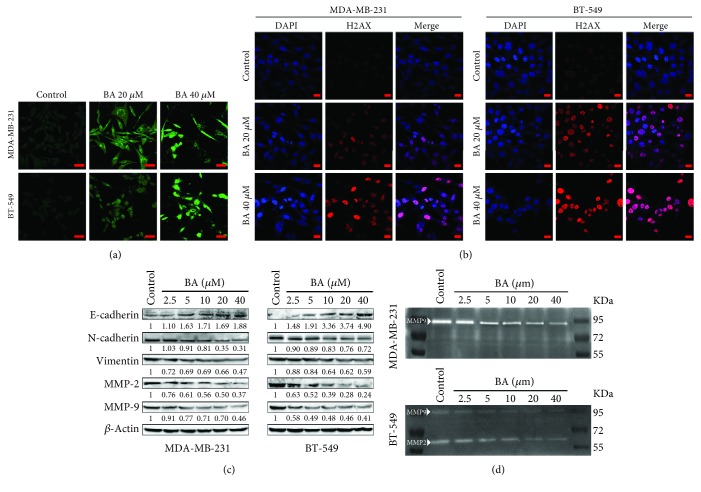
BA induces breast cancer cell DNA damage and inhibits EMT and MMPs. (a) TUNEL assay showed that BA induced MDA-MB-231 and BT-549 apoptosis (the scale bars indicate 50 *μ*m). (b) BA broke double-strand DNA in MDA-MB-231 and BT-549 cells, as represented by H2AX activation (the scale bars indicate 20 *μ*m). (c) BA reversed EMT in breast cancer cells, represented by a dose-dependent decrease in N-cadherin and vimentin and an increase in E-cadherin. MMP-2 and MMP-9 were also downregulated by BA treatment. (d) Gelatin zymography assay indicated that BA downregulated MMP-2 and MMP-9 secreted by breast cancer cells.

Previous studies demonstrated that BA induced cancer cell apoptosis and DNA damage directly. Similarly, our study also identified the role of BA in inducing MDA-MB-231 and BT-549 cell apoptosis via the TUNEL assay (Figure 2(a)). Besides, H2AX was activated in response to BA treatment, reflected by the presence of double-strand DNA breaks in breast cancer cells (Figure 2(b)). On the other hand, western blotting analysis also indicated that BA downregulated the levels of N-cadherin and vimentin as the mesenchymal markers, while increased E-cadherin which is an epithelial marker (Figure 2(c)), validating the EMT inhibition effects of BA in breast cancer cells. Since matrix metalloproteinases (MMPs) promote tumor metastasis by degrading the extracellular matrix (ECM), western blotting and gelatin zymography were used to measure the relative amounts of MMP-2 or MMP-9. The results indicated that BA significantly decreased the expression of MMP-2 and MMP-9 secreted by breast cancer cells (Figures 2(c) and 2(d)). All these findings further validated the activity of BA against breast cancer metastasis.

### 3.3. BA Suppresses Metastasis through *β*-Catenin-Mediated Aerobic Glycolysis

**Figure 3:**
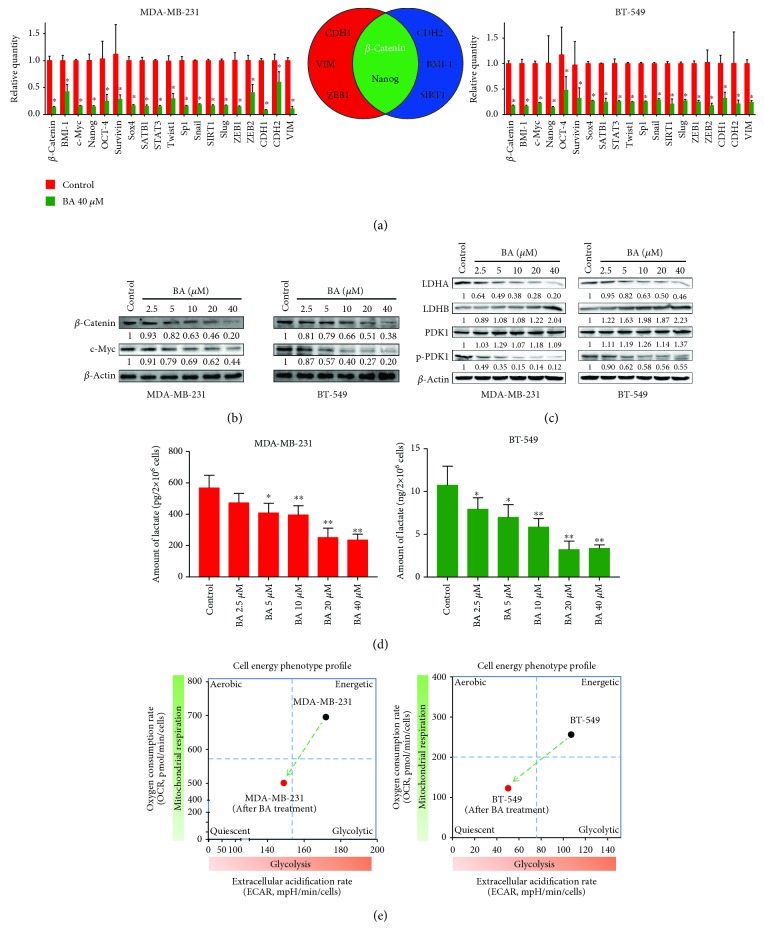
BA suppresses metastasis through *β*-catenin-mediated glycolysis. (a) The transcription levels of metastasis-related genes were screened by qPCR after BA treatment. Almost all genes were suppressed, among which *β*-catenin ranked as the most repressed gene in both cell lines (the results were obtained from triplicate experiments and were represented as mean values ± SD; ^∗^*P* < 0.05 as compared with control). (b) Western blotting further confirmed that *β*-catenin and its downstream target c-Myc were downregulated by BA in a dose-dependent manner. (c) BA dramatically attenuated the levels of glycolysis-related proteins including LDHA and p-PDK1/PDK, whereas LDHB was elevated due to its function of converting lactate into pyruvate. (d) BA reduced the lactate production of MDA-MB-231 and BT-549 cells in a dose-dependent manner (values were represented as mean ± SD; ^∗^*P* < 0.05 and ^∗∗^*P* < 0.01 as compared with control). (e) The cell energy phenotype was profiled by the extracellular flux analyzer. BA reduced ECAR and OCR values, keeping breast cancer cells in a relative quiescent energetic state.

Based on the above findings, further studies were needed to clarify the intrinsic association of BA in glycolysis and metastasis inhibition. Therefore, a panel of metastasis-related genes was selected to identify the most responsive gene influenced by BA using qPCR. Although the relative expression of all metastasis-associated genes was downregulated, *β-catenin* ranked first among the top five responsive genes in both MDA-MB-231 and BT-549 (Figure 3(a)). Interestingly, aberrant *β*-catenin accumulation and the activated downstream target gene of *c-Myc* are critical to cancer metastasis and metabolic alteration. We therefore assessed changes in their protein expression level in response to BA treatment. Western blotting results showed that BA dose-dependently downregulated the expression of *β*-catenin and c-Myc (Figure 3(b)). Meanwhile, the levels of glycolytic enzymes, including LDHA and p-PDK1/PDK1, were all decreased in a dose-dependent manner by BA. In contrast, LDHB that catalyzes the conversion of lactate to pyruvate was increased (Figure 3(c)). Consistently, lactate production in both MDA-MB-231 and BT-549 cells was significantly reduced following BA administration (Figure 3(d)), indicating that the glycolysis pathway may be inhibited by BA. What is more important, the cell energy phenotype of MDA-MB-231 and BT-549 was profiled by the extracellular flux analyzer. The results demonstrated that the extracellular acidification rate (ECAR), which reflects the glycolysis activity, was retarded following BA administration. Additionally, the oxygen consumption rate (OCR), which is a marker of mitochondrial respiration, was also decreased simultaneously (Figure 3(e)). Overall, these results implied that BA switched the cells from an energetic metabolic state to a relatively quiescent state, which might be closely correlated with the downregulation of the metabolic switch c-Myc. And *β*-catenin might be a panel point between the two hall markers of glycolysis and metastasis in tumor cells.

### 3.4. BA-Induced GRP78 Overexpression Restrains Aerobic Glycolysis of Breast Cancer Cells

Our previous study has uncovered that GRP78 is a molecular target for chemosensitizing effects of BA [31]. In the current study, it was also identified that GRP78 was the direct binding protein of BA in highly aggressive breast cancer cells by DARTS strategy. Following BA treatment at 0.1-100 *μ*M, a protected band around 70 kDa was presented (Supplementary [Supplementary-material supplementary-material-1]). The protected gel was subsequently identified as GRP78 by LC/MS analysis (Supplementary [Supplementary-material supplementary-material-1]).

**Figure 4:**
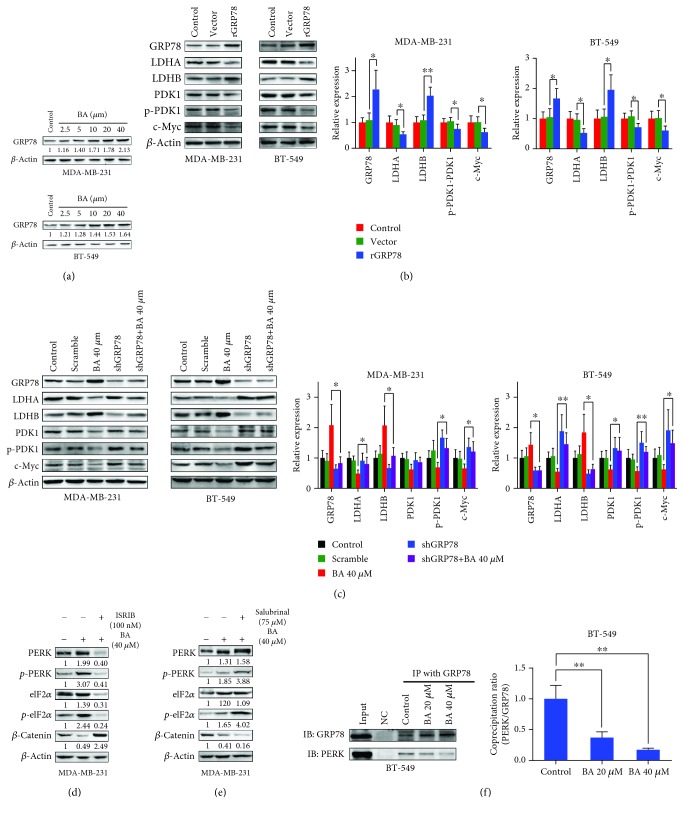
GRP78 overexpression suppresses aerobic glycolysis by activating PERK signaling to inhibit *β*-catenin. (a) Western blotting analysis verified that BA significantly enhanced GRP78 expression. (b) Overexpressed GRP78 led to the downregulation of c-Myc and subsequently decreased LDHA and p-PDK1/PDK1 but increased LDHB expression. (c) On the contrary, GRP78 knockdown reversed the inhibition of c-Myc, LDHA, and p-PDK1/PDK1 and the enhancement of LDHB induced by BA. (d) ISRIB (100 nM), the specific PERK inhibitor, inhibited eIF2*α* phosphorylation and reversed *β*-catenin inhibition induced by BA. (e) Like BA, salubrinal (75 *μ*M) inhibited eIF2*α* dephosphorylation and therefore downregulated *β*-catenin expression. (f) Coimmunoprecipitation assay revealed the binding of GRP78 and PERK, which was disrupted by BA in a dose-dependent manner (the results were obtained from triplicate experiments and were represented as mean values ± SD; ^∗^*P* < 0.05 and ^∗∗^*P* < 0.01).

Western blotting further confirmed that BA treatment significantly enhanced GRP78 expression in MDA-MB-231 and BT-549 cells (Figure 4(a)). To determine the functional relevance of GRP78 to BA inhibition of aerobic glycolysis in breast cancer, MDA-MB-231 and BT-549 cells were transfected with a recombinant GRP78 plasmid. Consistent with the pharmacological action of BA, overexpressed GRP78 attenuated the levels of glycolytic-related proteins, including LDHA, p-PDK1/PDK1, and c-Myc (Figure 4(b)). In contrast, GRP78 knockdown promoted the expression of glycolysis-related proteins and abolished the inhibitory effects of BA (Figure 4(c)), indicating the critical role of GRP78 in mediating the pharmacological action of BA. We next studied the possible mechanism by which GRP78 induction inhibited the switch activation of *β*-catenin/c-Myc for glycolysis. It is known that GRP78 would activate PERK signaling under ER stress and the activated PERK would phosphorylate eIF2*α*, resulting in the inhibition of protein translation. Therefore, we first verified that BA activated PERK signaling and in turn promoted phosphorylation of eIF2*α* (Supplementary [Supplementary-material supplementary-material-1]), which was also in line with our previous study that BA could activate ER stress apoptotic pathway. In addition, BA-induced downregulation of *β*-catenin was reversed by ISRIB, a specific inhibitor of PERK (Figure 4(d)). Conversely, salubrinal, a small molecule compound enhancing eIF2*α* phosphorylation, attenuated *β*-catenin expression (Figure 4(e)). Altogether, these results suggested that BA-induced downregulation of *β*-catenin was mediated by the GRP78/PERK/eIF2*α* pathway. Moreover, GRP78 and PERK could be coprecipitated in the highly aggressive breast cancer cells, and their interaction was interrupted following BA treatment in a dose-dependent manner (Figure 4(f), Supplementary [Supplementary-material supplementary-material-1]). These findings suggested that BA inhibited the *β*-catenin/c-Myc pathway by interrupting the binding between GRP78 and PERK and ultimately suppressed the glycolysis of breast cancer cells.

### 3.5. BA Suppresses Breast Cancer Metastasis *In Vivo*

**Figure 5:**
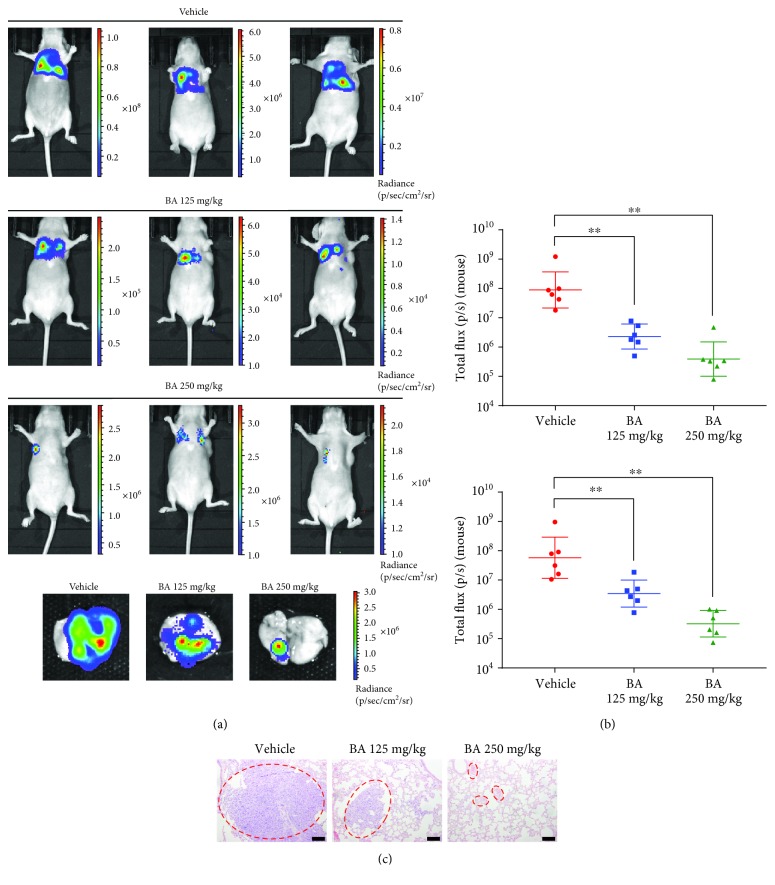
BA inhibits breast cancer lung colonization *in vivo*. (a) Bioluminescent imaging indicated that BA significantly reduced breast cancer cell lung colonization compared with vehicle-treated controls. (b) Logarithmic value of luminescent intensity after treatment with vehicle or BA (values represented as the mean ± SD, *n* = 6, ^∗∗^*P* < 0.01). (c) Hematoxylin and eosin staining demonstrated the reduction of lung metastatic lesions in BA-treated mice (the scale bars indicate 100 *μ*m).

Given the above results and the inhibitory effects of BA on cancer cell invasion and migration *in vitro*, a lung colonization model of breast cancer was established by injecting luciferase-labeled MDA-MB-231 cells through the lateral tail vein. The doses of BA were chosen according to the literatures [20] and our preliminary experiment. In fact, BA at 250 mg/kg did not induce observable morphological variations in primary organs of mice. In addition, BA at 250 mg/kg did not lead to noticeable changes on hematological, hepatic, and renal functions in mice (data not shown). After 4 weeks of treatment by BA, bioluminescent imaging demonstrated that BA significantly inhibited breast cancer cell colony growth in the lungs, representing as reduced luminescent intensity compared with vehicle-treated controls (Figures 5(a)–5(c)). In addition, hematoxylin and eosin staining confirmed that the pulmonary metastasis lesions were remarkably suppressed after BA administration (Figure 5(d)).

### 3.6. BA Retards Breast Cancer Lung Colonization by GRP78/*β*-Catenin/c-Myc Signaling

**Figure 6:**
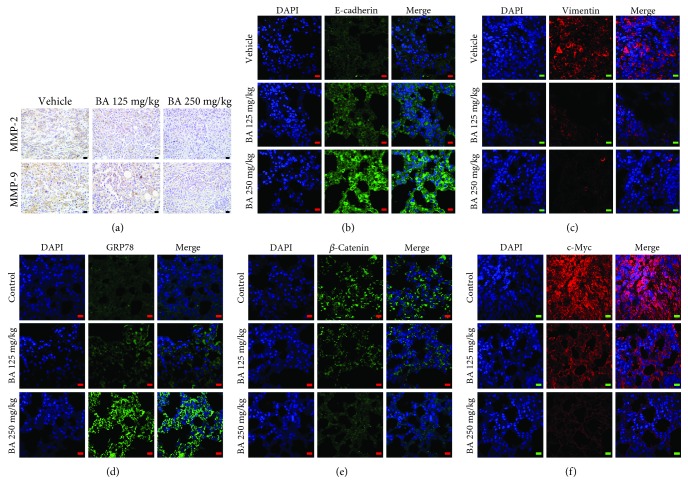
BA inhibits glycolysis signaling in metastatic lesions by targeting GRP78/*β*-catenin/c-Myc signaling. (a) MMP-2 and MMP-9 expressions were attenuated by BA administration in the lung colonization lesions (the scale bars indicate 20 *μ*m). (b, c) Immunofluorescence showed that BA increased E-cadherin expression in the lung tissue but reduced the expression of vimentin, suggesting that EMT in breast cancer was blocked by BA *in vivo* (the scale bars indicate 10 *μ*m). (d–f) Immunofluorescence demonstrated that BA significantly elevated GRP78 levels and decreased *β*-catenin/c-Myc signaling in lung metastatic lesions (the scale bars indicate 10 *μ*m).

Based on the *in vivo* results, immunohistochemistry analysis further revealed that MMP-2 and MMP-9 expressions were reduced in the lung colonization lesions after BA treatment, implying that BA might diminish the aggressiveness of breast cancer cells *in vivo* (Figure 6(a)). Immunofluorescence results further validated that BA suppressed the levels of vimentin and elevated E-cadherin expression (Figures 6(b) and 6(c)), confirming the blocking effect of BA in EMT process *in vivo*. The expressions of GRP78, *β*-catenin, and c-Myc were also detected in the lung colonization lesions after BA treatment. In line with previous *in vitro* findings, BA was found to enhance GRP78 expression and significantly inhibit *β*-catenin and c-Myc expression in the lung lesions (Figures 6(d)–6(f)), suggesting that the metastasis inhibition effects of BA were closely correlated with GRP78-mediated glycolysis inhibition. In conclusion, our results suggested that BA inhibits breast tumor metastasis *in vivo* and GRP78 might be the critical target of BA associating with its anticancer pharmacological action.

## 4. Discussion

Metabolic reprogramming is required for both malignant transformation and tumor development, including invasion and metastasis [35]. In this study, we found that BA restrained breast cancer metastasis by inhibiting aerobic glycolysis. Moreover, relatively high doses of BA applied *in vitro* and *in vivo* seemed to be acceptable due to its low toxicity. Also, numerous derivatives of BA have been validated with satisfactory anticancer efficacy. It is worth noting that a PEGylated derivative of BA possessed excellent water solubility of 160.2 mg/ml (about 750-fold higher than BA) and showed a high therapeutic index in a lung cancer xenograft model [36]. In addition, the structure-activity relationship analysis found that chemical modifications on the C-2 site enhanced the antitumor potency of BA [37]. However, few studies have examined why BA possesses selective cytotoxicity against cancer cells. Intriguingly, one study implied that BA might exert higher efficiency in low pH environments (around 6.8) [38]. Similar with this finding, our study also found that BA remarkably decreased cancer cell lactate production and the expression of glycolytic enzymes, which resulted in cell energy phenotype switching to a quiescent status. More importantly, our study identified GRP78, which is a glucose-regulated protein, as a direct interacting target of BA, further manifesting the central role of glucose metabolism in mediating the selective killing effects of BA in cancer cells. Actually, other pharmacological studies have also demonstrated that BA could reduce prostate cancer angiogenesis *via* inhibiting the HIF-1*α*/stat3 pathway [39]. A proteomic study also implied that a ROS-mediated pathway was the main target responsible for mediating the anticancer activities of BA [40]. All these findings suggested that the anticancer pharmacological mechanism of BA might be associated with stress signaling.

**Figure 7:**
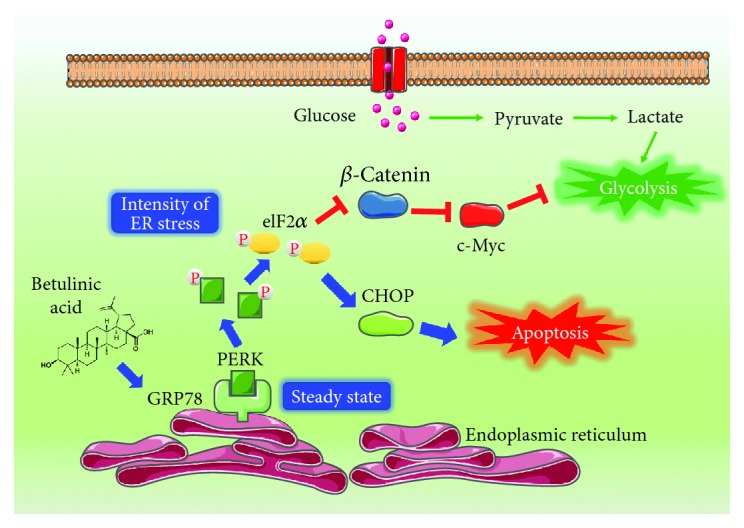
Diagram illustrating how BA inhibited glycolysis by targeting GRP78. BA interrupted the binding of GRP78 and PERK, which initiated ER stress, and subsequently activated eIF2*α* phosphorylation, resulting in *β*-catenin inhibition and c-Myc-mediated aerobic glycolysis. Meanwhile, the ER stress apoptotic pathway was triggered.

Aberrant cellular stress is another hallmark of cancer [41]. The rapid proliferation of cancer cells creates a relatively nutrient-starved microenvironment, causing cancer cells to adapt to this “stressful” condition by activating ER stress signaling [42]. Emerging evidence has demonstrated that ER stress influences cellular metabolism through various mechanisms. ER stress is known to stimulate lipogenesis through the unfolded protein response (UPR), thereby providing lipids for ER expansion. Mechanistic studies have revealed that ER stress can promote fatty acid and cholesterol biosynthesis through two major transcriptional regulators: SREBP1 and SREBP2 [43, 44]. In addition, PERK, as the sensor of ER stress, has also been reported to be significant for lipogenic tissue development, since PERK knockout impairs mouse mammary gland lipogenesis during pregnancy, which leads to a reduction of free fatty acid in milk [45]. Furthermore, ER stress would facilitate physical coupling and calcium transfer from ER to mitochondria, resulting in augmented mitochondrial respiration and bioenergetics, thereby enhancing adaptive ATP production [46]. However, it is still unclear how ER stress affects cancer aerobic glycolysis. In the present study, we reported that the ER stress protein GRP78 inhibited aerobic glycolysis by promoting PERK activation, which impaired *β*-catenin production and consequently inhibited downstream c-Myc-mediated glycolysis (Figure 7). Our study proposes a regulatory relationship between ER stress and glycolysis metabolism in tumor cells and highlights that targeting ER stress to inhibit cancer aerobic glycolysis might be a novel strategy for cancer therapy.

GRP78, a biomarker of ER stress, is highly expressed in multiple malignancies. Additionally, GRP78 has also been reported to play an important role in cancer metastasis. For example, GRP78 was identified as a potential diagnostic biomarker for the early detection of melanoma metastasis [47]. High expression of GRP78 was also detected in the metastatic phenotype of prostate cancer [14], hepatocellular carcinoma [48], and esophageal squamous cell carcinoma [49]. In our study, the precise target of BA was identified as GRP78 by using DARTS technology. However, the antimetastasis effects of BA were found to be correlated with enhanced GRP78 expression. Meanwhile, GRP78 overexpression could suppress the expression of glycolytic proteins including LDHA, PDK1, and c-Myc. These results indicated that GRP78 overexpression could inhibit cancer glycolysis. Our findings kept consistency with the report by Li et al. [17], which found that GRP78 overexpression induced a decline in the PKM2 level in colorectal cancer cells. What is more important, the overexpression of GRP78 also downregulated the LDHA mRNA expression, accompanied by reduced lactic acid secretion. However, some studies also reported that GRP78 overexpression could enhance glycolysis activity. For example, Miharada et al. found that Cripto/GRP78 signaling could improve glycolysis activity in hematopoietic stem cells by regulating HIF-1*α* [50, 51]. As well known, GRP78, as the stress-responsive chaperone, plays dual roles in cancer initiation and development. To adapt to the hypoxic and glucose-deprivation microenvironment in tumor tissues, GRP78 was activated to degrade the unfolded protein to satisfy the nutrient requirements of cancer cells. Therefore, GRP78 upregulation was usually found in multiple cancer cells and associated with drug resistance and metastasis and angiogenesis. However, when GRP78 was overactivated, it will trigger the ER stress apoptotic pathway and induce cell death. Furthermore, the activation of the PERK/eIF2*α* pathway in the ER stress signaling would result in the inhibition of *β*-catenin translation, which led to c-Myc-mediated glycolysis suppression (Figure 7). Since the expression of GRP78 is significantly higher than normal cells, further exogenous overexpression of GRP78 would make cells to be in an excessive stressful situation and towards apoptosis finally. Therefore, selectively triggering ER stress by targeting GRP78 in cancer cells might be a promising approach for future drug discovery. Although BA demonstrated selective killing effects in cancer cells, its specificity towards GRP78 and targeting for drug delivery still needs to be investigated.

## 5. Conclusions

In conclusion, this study uncovered the mechanism of BA in inhibiting breast cancer metastasis by targeting GRP78 to trigger ER stress signaling, subsequently suppressing aerobic glycolysis. Our results shed new light on BA antimetastasis through suppressing aerobic glycolysis and also highlight GRP78 as a potential regulatory target for tumor glucose metabolism.

## Data Availability

The data used to support the findings of this study are available from the corresponding author upon request.

## Supplementary Materials

Supplementary Materials Supplementary Figure 1: identification of GRP78 as a target of BA in highly aggressive breast cancer cells. (A) After treatment with BA, a protected band around 70 kDa emerged by Coomassie blue staining. The black arrow indicates the protected band. (B) The most probable protein was identified as GRP78 through mass spectroscopy analysis. The representative peptide mass fingerprints were shown. Supplementary Figure 2: BA promotes the dissociation of GRP78 and PERK to activate the PERK signaling of ER stress. (A) PERK signaling and eIF2*α* phosphorylation were provoked by BA in a dose-dependent manner. (B) The binding between GRP78 and PERK was found in MDA-MB-231 cells by coimmunoprecipitation assay, which was attenuated after BA treatment (the results were obtained from triplicate experiments and were represented as mean values ± SD; ^∗^*P* < 0.05, ^∗∗^*P* < 0.01).
