# Supplementary material for: Betulinic Acid Suppresses Breast Cancer Metastasis by Targeting GRP78-Mediated Glycolysis and ER Stress Apoptotic Pathway
Source: Oxid Med Cell Longev. 2019 Aug 19;2019:8781690. doi: 10.1155/2019/8781690 (PMC6721262; doi:10.1155/2019/8781690)
Supplement: Supplementary Materials — Supplementary Figure 1: identification of GRP78 as a target of BA in highly aggressive breast cancer cells. (A) After treatment with BA, a protected band around 70 kDa emerged by Coomassie blue staining. The black arrow indicates the protected band. (B) The most probable protein was identified as GRP78 through mass spectroscopy analysis. The representative peptide mass fingerprints were shown. Supplementary Figure 2: BA promotes the dissociation of GRP78 and PERK to activate the PERK signaling of ER stress. (A) PERK signaling and eIF2α phosphorylation were provoked by BA in a dose-dependent manner. (B) The binding between GRP78 and PERK was found in MDA-MB-231 cells by coimmunoprecipitation assay, which was attenuated after BA treatment (the results were obtained from triplicate experiments and were represented as mean values ± SD; ∗P < 0.05, ∗∗P < 0.01). [file 8781690.f1.pdf]

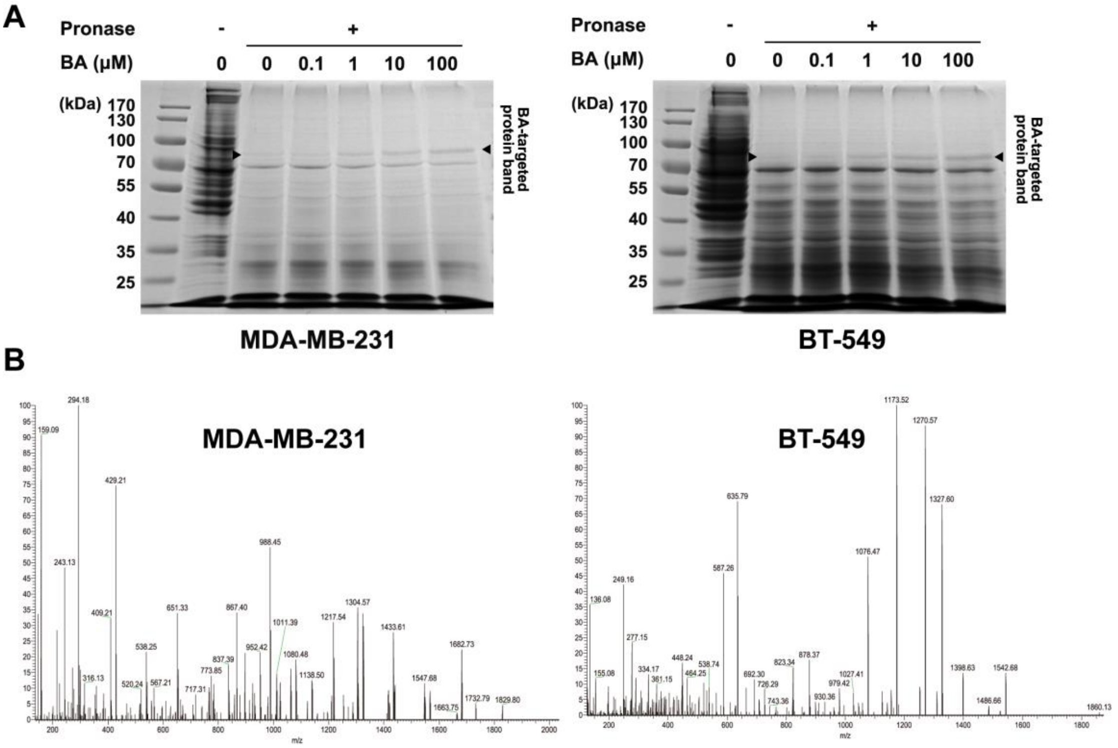

Supplementary Figure 1. Identification of GRP78 as a target of BA in highly aggressive breast cancer cells. (A) After treatment with BA, a protected band around 70 kDa emerged by Coomassie Blue staining. The black arrow indicates the protected band. (B) The most probable protein was identified as GRP78 though mass spectroscopy analysis. The representative peptide mass fingerprints were showed.

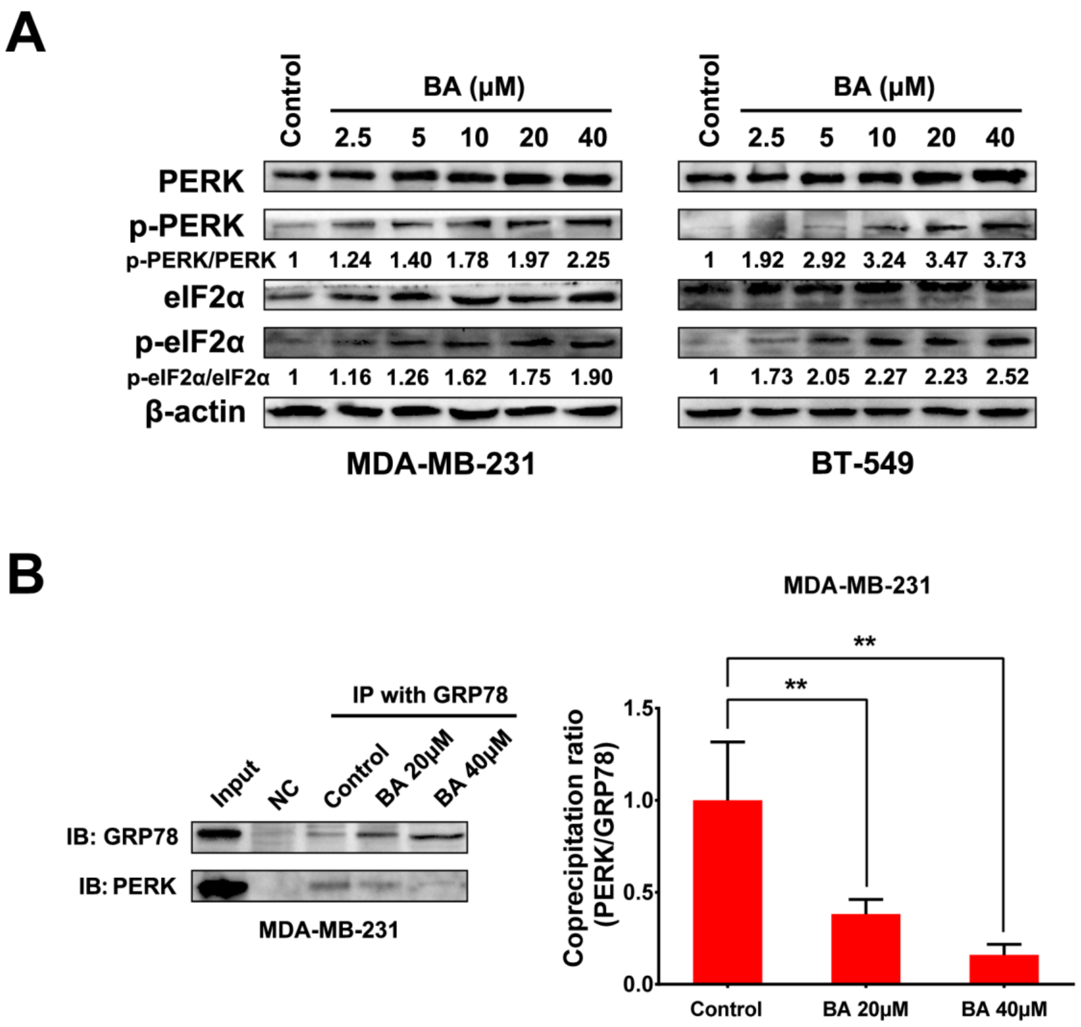

Supplementary Figure 2. BA promotes the dissociation of GRP78 and PERK to activate the PERK signaling of ER stress. (A) PERK signaling and eIF2α phosphorylation were provoked by BA in a dose-dependent manner. (B) The binding between GRP78 and PERK was found in MDA-MB-231 cells by coimmunoprecipitation assay, which was attenuated after BA treatment (The results were obtained from triplicate experiments and were represented as mean values ± SD. *$P < 0.05$, **$P < 0.01$).
